# Mortality predictors in patients with suspected sepsis in the emergency department of a tertiary care hospital: a retrospective cohort study

**DOI:** 10.1186/s12245-024-00655-9

**Published:** 2024-06-17

**Authors:** João P. M. Bidart, Regis G. Rosa, Marina Bessel, Luana G. Pedrotti, Luciano Z. Goldani

**Affiliations:** 1 Emergency Department, Moinhos de Vento Hospital, 910, Ramiro Barcelos Street, Porto Alegre, Zip code 90035-001 Brazil; 2 Internal Medicine Department, Moinhos de Vento Hospital, Ramiro Barcelos, 630, Porto Alegre, 90035- 001 Brazil; 3 Proadi Social Responsability, Moinhos de Vento Hospital, Ramiro Barcelos, 910, Porto Alegre, 90035- 000 Brazil; 4 ⁵Section of Infectious Diseases, Hospital de Clínicas de Porto Alegre, Universidade Federal do Rio Grande do Sul, Ramiro Barcelos 2400, Porto Alegre, 90035-003 Brazil

**Keywords:** Emergency department, Sepsis, Septicemia, Quick sofa, Intensive care unit

## Abstract

**Background:**

Sepsis remains a worldwide major cause of hospitalization, mortality, and morbidity. To enhance the identification of patients with suspected sepsis at high risk of mortality and adverse outcomes in the emergency department (ED), the use of mortality predictors is relevant. This study aims to establish whether quick sofa (qSOFA) and the severity criteria applied in patients with suspicion of sepsis in a monitored ED are in fact predictors of mortality.

**Methods:**

We performed a retrospective cohort study among adult patients with suspicion of sepsis at the ED of a tertiary care hospital in Brazil between January 1st, 2019 and December 31, 2020. All adult patients (ages 18 and over) with suspected sepsis that scored two or more points on qSOFA score or at least one point on the severity criteria score were included in the study.

**Results:**

The total of patients included in the study was 665 and the average age of the sample was 73 *±* 19 years. The ratio of men to women was similar. Most patients exhibited qSOFA ≥ 2 (58.80%) and 356 patients (53.61%) scored one point in the severity criteria at admission. The overall mortality rate was 19.7% (131 patients) with 98 patients (14.74%) having positive blood cultures, mainly showing Escherichia coli as the most isolated bacteria. Neither scores of qSOFA nor the severity criteria were associated with mortality rates, but scoring any point on qSOFA was considered as an independent factor for intensive care unit (ICU) admission (qSOFA = 1 point, *p* = 0.02; qSOFA = 2 points, *p* = 0.03, and qSOFA = 3 points, *p* = 0.04). Positive blood cultures (RR, 1.63;95% CI, 1.10 to 2.41) and general administration of vasopressors at the ED (RR, 2.14;95% CI, 1.44 to 3.17) were associated with 30-day mortality. The administration of vasopressors at the ED (RR, 2.25; CI 95%, 1.58 to 3.21) was found to be a predictor of overall mortality.

**Conclusions:**

Even though an association was found between qSOFA and ICU admission, there was no association of qSOFA or the severity criteria with mortality. Therefore, patients with a tendency toward greater severity could be identified and treated more quickly and effectively in the emergency department. Further studies are necessary to assess novel scores or biomarkers to predict mortality in sepsis patients admitted to the ED’s initial care.

## Introduction

Sepsis remains a worldwide major cause of hospitalization, mortality, and morbidity leading to a considerable healthcare concern according to the World Health Organization (WHO) [1, 2]. In 2017, about 48.9 million cases of sepsis were recorded in the world, leading to an estimated number of 11.0 million related deaths, a number that represents about 19.7% of all global deaths in the same period [3].

Quick sofa (qSOFA) was introduced as a bedside or triage tool to facilitate the identification of patients with suspicion of sepsis [4]. This new measure incorporates three variables: altered mental status, systolic blood pressure (SBP) of 100 mmHg (or below), and respiratory rate of 22/min (or higher) [5]. In addition, some studies showed that qSOFA scores has high specificity but low sensitivity for prediction in hospital mortality in patients with sepsis or suspicion of sepsis outside the intensive care unit (ICU) [6, 7]. In the emergency department (ED) scenario, patients with suspicion of sepsis presenting qSOFA ≥ 2 at triage are expected to represent a population of higher risk of mortality, pointing this tool as a rapid, simple, and inexpensive resource for the early identification of patients that are at risk for negative outcomes [8].

Other predictors of mortality in patients with sepsis described in the literature are mottled skin, bacteremia, and tachycardia (heart rate above 120 beats) [9–13]. Several studies have shown an increase in mortality in patients that enter the triage with high scores on the mottling score [10, 11, 14, 15]. Other studies have demonstrated that patients with positive blood cultures present higher mortality rates when compared to individuals with negative blood cultures [12, 13]. Based on these predictors, a new score called severity criteria, composed of three variables (mottled skin at presentation, tachycardia > 120 bpm, and shivering suggesting bacteremia at presentation) was created by the studied institution and used as part of the institutional sepsis protocol with the objective to enhance the sensitivity of qSOFA and then possibly correlate it with the main adverse outcomes.

The ED is a place of high flow of patients afflicted with diverse and complex pathologies. Both emergency physicians and nursing staff need simple, quick, and practical tools to apply in emergency triage in order to detect patients in severe conditions and at high risk of death. In order to improve the ability to identify patients with suspected sepsis in a high possibility of mortality and adverse outcomes in the ED, mortality predictors are applicable, including qSOFA and the severity criteria with the expected outcomes. The objective of this study is to establish whether qSOFA and the severity criteria applied in patients with suspicion of sepsis in a monitored ED are in fact predictors of mortality.

## Methods

We performed a retrospective cohort study among adult patients with suspicion of sepsis at the ED of a tertiary care hospital in Brazil between January 1st, 2019, and December 31, 2020. The hospital has a total of 485 beds with 113 in the ICU and 372 in the ward. The ED also has 34 additional beds for patients admitted to this unit. The institutional protocol for sepsis was applied for all adults (ages 18 and over) admitted at the triage of the hospital’s ED with symptoms suggestive of infection (such as fever, respiratory abnormalities, abdominal pain/diarrhea, urinary symptoms, headache, alteration of mental status, skin tissue alterations, among other features). Patients that scored two or more points on the qSOFA score or at least one point on the severity criteria score were considered as potentially septic and were evaluated by a physician to discard alternative diagnoses. Severity criteria ranges from 0 to 3, considering patients with shivering at presentation, mottled skin at presentation and tachycardia ≥ 120 beats/min at presentation, each one being counted as 1 point in the scale. Although qSOFA has been widely studied and validated in the literature as a screening and prognostic tool, the severity criteria were developed by a consensus of specialists from the institution in order to increase the sensitivity of qSOFA in predicting the mortality of patients with suspected sepsis. Whenever the diagnosis of sepsis was considered, the patients were screened for inclusion in the study. The exclusion criteria included: (*i*) patients in palliative care defined by the non-institution of invasive measures such as hemodialysis, central venous access, intubation or resuscitation; (*ii*) patients diagnosed with COVID-19;(*iii*) patients whose access to medical records were restricted; (*iv*) patients that were diagnosed with other conditions and (*v*) pregnancy.

The primary outcome was overall mortality and 30-day mortality. Secondary outcomes were: (*i*) length of stay in hospital and ICU admission; (*ii*) discrimination of the main microorganisms found in positive blood cultures; and (*iii*) determination of the impact of antibiotic (ATB) initiation on mortality rates.

In order to proceed with the investigation, the following data were collected from the patients’ electronic medical records: age, gender, qSOFA, and the severity criteria scores, as well as qSOFA components such as SBP ≤ 100 mm Hg, respiratory rate ≥ 22 breaths per minute, and altered mental state (Glasgow Coma Scale ≤ 14). The severity criteria components described were mottled skin, tachycardia > 120 bpm, and shivering suggesting bacteremia at presentation. Each component represents one point in each score, with a maximum of three points for both qSOFA and the severity criteria.

Platelets, lactate, creatinine, bilirubin, and blood cultures were collected preferably within one hour from the arrival of patients in the ED. The blood culture bottles used were BD BACTEC (Becton, Dickinson and Company, Sparks, USA).To consider positive blood cultures, common skin contaminants like coagulase-negative *staphylococci*, *Bacillus Species*, *Corynebacterium* species, *micrococci*, and *Propionibacterium* species were disregarded unless they were cultured from two or more blood cultures [16]. Additional variables such as use of vasopressors and time of ATB administration were also collected. Time to ATB was defined as the time between the patient’s arrival in the ED and the time the ATB was administered. The aforementioned possible outcomes (30-day mortality, ICU admission, length of stay in hospital, and overall mortality) were evaluated. Missing values were assumed to be within the normal range (i.e., the value assigned was 0).

Considering that mortality in sepsis in this hospital is around 17% and using a sample power of 90% with a significance level of 0.05, The estimated minimum sample size was 217 patients. The categorical variables in this study were presented by absolute frequencies and percentage, while the continuous variables were described by means and standard deviation or medians and interquartile range. In order to compare proportions, we employed the Chi-Square test. For continuous variables, the Mann-Whitney test was chosen. Multivariable analysis was also performed for each outcome through the Poisson regression, with robust variance using the hierarchical model. The variables that were included in the model were sex, age, different qSOFA points, different severity criteria points, time to ATB, lactate, blood cultures, creatinine, platelets, and administration of vasopressor. The statistical analysis was performed in SAS© (Statistical Analysis System, SAS Institute Inc., Cary, N.C.), version 9.4, and the R Software (version 4.0.3) was used in Receiving Operating Characteristic (ROC) curves to measure the accuracy of the scores. For all the analysis, the level of significance considered was 5%.

## Results

**Fig. 1:**
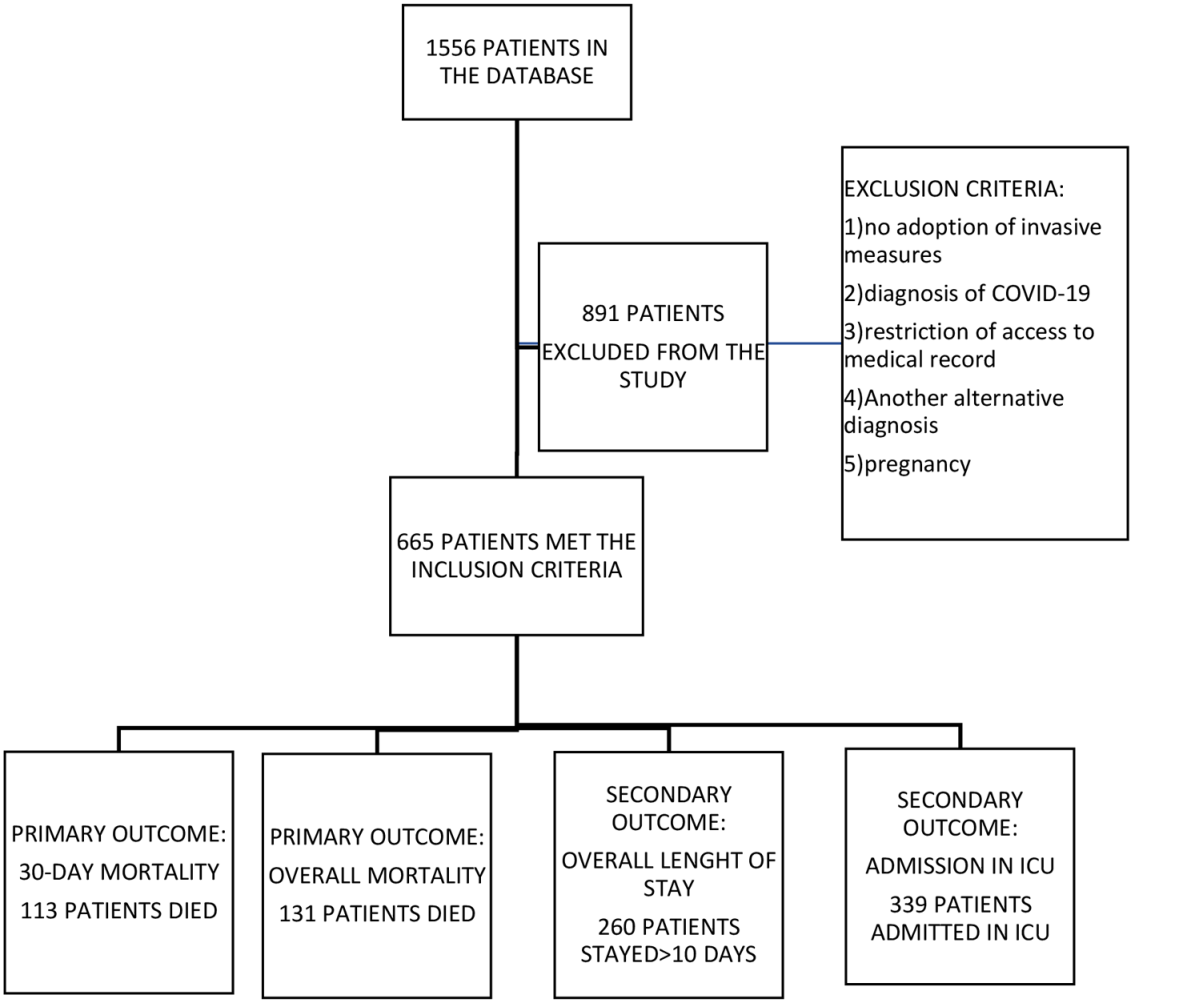
Flowchart of the study with the main outcomes

1,556 patients were admitted and assessed for infection at the ED triage. Of these, 665 patients were eligible for suspicion of sepsis during the period between January 1st, 2019, to December 31, 2020. Figure 1 shows the flowchart of the study with the number of patients included along with the results of the main outcomes found.

The demographic, clinical, laboratory, and main outcomes of the population in the study are described in Table 1. The average age in the sample was 73.36 years old and 75% of the patients were elderly (≥ 65 years). The ratio of men to women was similar. The predominant site of infection found in the study was respiratory (49.77%) such as bronchopneumonia; followed by urinary tract infections (23.01%) such as cystitis and pyelonephritis; abdominal infections (12.33%) such as intra-abdominal abscesses; colitis, diverticulitis and peritonitis; and skin infections (3,01%), such as erysipelas, cellulitis and abscesses.

**Table 1:** Characteristics of the patients with suspicion of sepsis

Characteristic^a^	Patient data
**Sex (No. [%])**	
Female	344(51.73)
Male	321(48.27)
Age (mean [SD]) (yr)	73.3(19.19)
**No. (%)**	
18–64 ≥ 65	163 (24.51) 502 (75.49)
Glasgow Coma Scale score ≤ 14(No. [%])^b^	208(32.50)
SBP ≤ 100 mmHg (No. [%])	345(51.88)
Respiratory rate ≥ 22 breaths/min (No. [%])	485(72.93)
**qSOFA points (No. [%])**	
0 1 2 3	71(10.68) 203(30.53) 334(50.23) 57(8.57)
qSOFA ≥ 2 points (No. [%])	391(58.80)
Shivering (No. [%])	76(11.43)
Mottled skin (No. [%])	84(12.63)
Tachycardia ≥ 120 beats/min (No. [%])	310(46.62)
**Severity Criteria points (No. [%])** ^c^	
0 1 2 3	253(38.10) 356(53.61) 51(7.68) 4(0.60)
Vasopressor at ED (No. [%])	138(20.75)
**Site of infection (No. [%])**	
Respiratory Urinary Abdominal Cutaneous Febrile Neutropenia Unknown More than one site Another ^d^	331(49.77) 153(23.01) 82(12.33) 20(3.01) 13(1.95) 39(5.86) 19(2.86) 8(1.20)
**Laboratory results (median [IQR])**	
Platelets (10³ /µL) Bilirubin(mg/dL) Creatinine (mg/dL) Lactate(mmol/L)	208.50(153.50-263.50) 0.50(0.34–0.76) 1.16(0.87–1.71) 2.06(1.40–3.06)
**Blood cultures (No. [%])** ^f^	
Negative Positive Contaminant	505(75.94) 98(14.74) 60(9.02)
Time to ATB (mean [SD]) (minutes) ^g^ No. (%)	37.43(48)
< 30 30–60 > 60	228(34.39) 388(58.52) 47(7.09)
30-Day mortality (No. [%])	113(16.99)
ICU admission (No. [%])	339(50.98)
Overall lenght of stay (median [IQR]) (days)	10(6–17)
Overall mortality (No. [%])	131(19.7)

(^a^) *Abbreviation* SD, standard deviation; IQR, interquartile range; SBP, systolic blood pressure; qSOFA, quick Sepsis-related Organ Failure Assessment; ED, emergency department; ATB, antibiotic; ICU, intensive care unit. (^b^) Missing value 25. (^c^) Missing value 1. (^d^) Other sites of infection were considered central nervous system, catheter and osteoarticular; (^e^) The number of patients for those with platelets is 660; creatinine values, 663; bilirubin, 601; lactate values, 656. (^f^) The number of patients for those with blood cultures is 662. (^g^) Missing value 2

Most patients presented qSOFA ≥ 2 at admission, representing a group of patients with high scores and with a tendency towards higher mortality, which suggests that patients were in a severe condition at admission. In addition, 411 patients scored at least one point on the severity criteria, with most scoring only one point when they arrived at the emergency triage (53.61%).

When the patients were recognized by the protocol as potentially septic, the mean time of ATB start was 37.43 min. Patients who received the ATB were divided into three groups according to the time of administration. Approximately 34% of the patients received antimicrobial therapy within 30 min, 58.52% started between 30 and 60 min, and only 7% received it above one hour. About 17% of the patients included in the study died within 30 days, with almost half being admitted in the ICU, having around a 10-days length of stay in hospital.

Among the 665 patients included in the study, 98 patients exhibited positive blood cultures (14.74%), 505 exhibited negative blood cultures (75.94%), and 60 presented blood cultures that were considered contaminants (9.02%). Table 2 displays the bacteria detected by blood cultures in the entire cohort of patients. Among the 98 positive blood cultures, 56 were gram-negative bacteria (57.14%), 26 were gram-positive bacteria (26.53%). The most common gram-negative isolated in blood cultures was 39 *Escherichia coli* (39.80%), nine *Klebsiella pneumoniae* (9.18%), four *Pseudomonas aeruginosa* (4.08%), three *Proteus Mirabilis* (3.06%), with only one case of *Neisseria meningitidis* (1.02%) being detected. The most common gram-positive bacteria isolated were eight *Staphylococcus aureus* (8.16%), four *Enterococcus faecalis* (4.08%), three *Streptococcus pneumoniae* (3.06%), two *Staphylococcus epidermidis* (2.04%) that were not considered as contaminants, and nine (9.18%) other *Streptococcus* species, except for *Streptococcus pneumoniae*. Eight of the positive blood cultures were considered as other bacteria (8.16%), and eight as polymicrobial flora (8.16%).

**Table 2:** Bacteria isolated in positive blood cultures^a^

Gram-positive	No. (%)
*Staphylococcus aureus*	8 (8.16)
*Enterococcus faecalis*	4(4.08)
*Streptococcus pneumoniae*	3(3.06)
*Staphylococcus epidermidis*	2(2.04)
Other *Streptococcus* species^b^	9(9.18)
**Gram negative**	**No. (%)**
*Escherichia coli*	39(39.80)
*Klebsiella pneumoniae*	9(9.18)
*Pseudomonas aeruginosa*	4(4.08)
*Proteus mirabilis*	3(3.06)
*Neisseria meningitidis*	1(1.02)
Other^c^	8(8.16)
Polymicrobial flora^d^	8(8.16)

^(a)^ Missing values 3

^(b)^S. gallolyticus, S. lutetiensis, S. pyogenes, S. anginosus, S. agalactiae, S. dysgalactiae

^(c)^ Aeromonas sp, Achromobacter xylosoxidans, Acinetobacter baumanii, Corynebacterium striatum, Burkholderia cepacia, Gemella haemolysans, Serratia marcescens

^(d)^ considered as the association between more than one bacterium found in two distinct blood cultures. The bacteria found in this category were: *Enterobacter sp*, *Staphylococcus epidermidis*, *Pseudomonas aeruginosa, Escherichia coli, Morganella morganii, Klebsiella aerogenes, Streptococcus anginosus, Enterococcus gallinarum, Streptococcus oralis, Staphylococcus aureus*, and *Klebsiella pneumoniae*

Table 3 shows predictors of 30-day and overall mortality in patients with suspicion of sepsis. The reported number of deaths in 30 days was 113 (17%), while overall deaths were 131(19.7%). The univariate analysis has shown that age ≥ 65 years, three points on qSOFA, shorter times before ATB administration, and higher values of lactate were risk factors associated with higher mortality in 30 days. The same applies to other variables such as three points on qSOFA and lactate values. Positive blood cultures (RR, 1.63;95% CI, 1.10 to 2.41) and administration of vasopressors in the ED (RR, 2.14;95% CI, 1.44 to 3.17) featured as independent factors of 30-day mortality in the multivariate analysis.

When the outcome analyzed was overall mortality, age ≥ 65 years, three points on qSOFA at the triage, shorter times before the administration of ATBs (*p* = 0.02), higher values of lactate (*p* = 0.04), and positive blood cultures(*p* = 0.002) were only statistically significant in the univariate analysis. Administration of vasopressors at the ED (RR, 2.25; CI 95%, 1.58 to 3.21) was the only variable that was considered an independent risk factor in the non-survival group in the multivariate analysis.

**Table 3:** Predictors of 30-day mortality and overall mortality in patients with suspicion of sepsis according to regression analysis

Variables^a^	**30-day mortality**		Univariate analysis		Multivariate analysis	
	Yes (No. =113)	No (No. =552)	RR (95% CI)^b^	p value	RR (95% CI)	p value
**AGE (No. [%]) (yr)**						
≥65	94 (83.19)	408 (73.91)	1.61(1.01–2.55)	0.04	1.32(0.82–2.13)	0.26
**qSOFA points (No. [%])** ^c^						
1 2 3	29 (25.66) 59 (52.21) 18 (15.93)	174(31.52) 275(49.82) 39(7.07)	1.45(0.66–3.16) 1.79(0.85–3.76) 3.20(1.44–7.13)	0.35 0.12 0.004	0.98(0.46–2.08) 1.15(0.56–2.36) 1.72(0.78–3.77)	0.95 0.71 0.18
Time to ATB (median [IQR]) (minutes)^c^	31(24–40)	35(26–46)	0.99(0.97-1.00)	0.047	0.99(0.98–1.01)	0.25
Lactate (median [IQR]) (mmol/L)	2.48(1.65–4.77)	2.00(1.34–2.81)	1.02(1.00-1.05)	0.03	1.02(1.00-1.04)	0.06
Positive blood cultures (No. [%])	27 (23.89)	70 (12.68)	2.07(1.40–3.06)	< 0.001	1.63(1.10–2.41)	0.02
Vasopressor at ED (No. [%])	47 (41.59)	91 (16.49)	2.72(1.99–3.76)	< 0.001	2.14(1.44–3.17)	< 0.001
**Variables** ^a^	**Overall mortality**		**Univariate analysis**		**Multivariate analysis**	
	Yes(n = 131)	No(n = 534)	RR (95% CI)^b^	p value	RR (95% CI)	p value
**AGE (No. [%]) (yr)**						
≥65	108(82.44)	394(73.78)	1.52(1.01–2.31)	0.04	1.27(0.82–1.95)	0.28
**qSOFA points (No. [%])** ^c^						
1	35(26.72)	168(31.46)	1.36(0.69–2.69)	0.38	0.92(0.47–1.81)	0.81
2	68(51.91)	266(49.81)	1.61(0.84–3.07)	0.15	1.03(0.55–1.95)	0.92
3	19(14.50)	38 (7.12)	2.63(1.29–5.36)	0.007	1.41(0.70–2.84)	0.33
Time to ATB (median [IQR]) (minutes)^c^	31(23–40)	35(26–46)	0.99(0.97-1.00)	0.02	0.99(0.98-1.00)	0.12
Lactate (median [IQR]) (mmol/L)	2.41(2.59–4.25)	2(1.34–2.79)	1.02(1.00-1.04)	0.04	1.02(1.00-1.04)	0.10
Positive blood cultures (No. [%])	28 (21.37)	69(12.92)	1.76(1.21–2.54)	0.002	1.38(0.95–1.99)	0.09
Vasopressor at ED (No. [%])	54 (41.22)	84 (15.73)	2.68(2.00-3.59)	< 0.001	2.25(1.58–3.21)	< 0.001

^(a)^*Abbreviation* IQR, interquartile range; qSOFA, quick Sepsis-related Organ Failure Assessment; ATB, antibiotic; ED, emergency department

^(b)^ RR, relative risk; CI, confidence interval

^(c)^ The number of patients that had antibiotic administered is 663

Table 4: shows the association among the variables studied and the secondary outcomes: overall length of stay and ICU admission in patients with suspicion of sepsis admitted in the ED. The univariate analysis showed that patients with two points on qSOFA at presentation had longer periods (> 10 days) of length of stay in hospital and patients with one point on the severity score at presentation were associated with shorter periods (≤ 10 days). The feature age ≥ 65 years (*p* < 0.001; RR, 1.52;95% CI, 1.19–1.93) was significantly associated with longer periods of stay in hospital along with administration of vasopressors in the ED (RR, 1.31;95% CI, 1.10 to 1.56) in the multivariate analysis. Lower values of platelets (RR, 1.01; CI 95%, 1.01 to 1.02) at presentation were also significantly associated with shorter periods of stay, but in both groups the median values were in the normal range.

**Table 4:** Predictors of overall lenght of stay and ICU admission in patients with suspicion of sepsis according to regression analysis

Variables^a^	Overall lenght of stay			
	**Univariate analysis**		**Multivariate analysis**	
	RR (95% CI)^b^	p value	RR (95% CI)	p value
**Age (yr)**				
≥ 65	1.56(1.24–1.98)	< 0.001	1.52(1.19–1.93)	< 0.001
**qSOFA points**				
1	1.38(0.97–1.99)	0.07	1.33(0.94–1.88)	0.10
2	1.47(1.04–2.07)	0.03	1.19(0.82–1.73)	0.36
3	1.51(1.00-2.28)	0.05	1.19(0.77–1.83)	0.43
**Severity Criteria points**				
1	0.84(0.71–0.99)	0.04	0.90(0.72–1.13)	0.36
2	0.90(0.66–1.23)	0.52	0.97(0.69–1.35)	0.85
3	0.96(0.36–2.57)	0.93	1.02(0.49–2.13)	0.95
Platelets	1.01(1.01–1.02)	< 0.001	1.01(1.01–1.02)	0.001
Vasopressor at ED	1.31(1.10–1.55)	0.002	1.31(1.10–1.56)	0.002
Variables^a^	**ICU Admission**			
	**Univariate analysis**		**Multivariate analysis**	
	RR (95% CI)^b^	p value	RR (95% CI)	p value
**qSOFA points**				
1	1.64(1.13–2.38)	0.009	1.59(1.09–2.33)	0.02
2	1.71(1.19–2.46)	0.003	1.54(1.04–2.26)	0.03
3	0.74(1.41–3.11)	< 0.001	1.54(1.03–2.31)	0.04
**Severity Criteria points**				
1	1.09(0.93–1.29)	0.29	1.08(0.91–1.29)	0.39
2	1.15(0.87–1.52)	0.33	1.09(0.82–1.44)	0.55
3	2.09(1.84–2.38)	< 0.001	1.47(0.95–2.28)	0.09
Positive blood cultures	1.49(1.26–1.76)	< 0.001	1.17(1.01–1.36)	0.04
Creatinine	1.02(1.01–1.03)	0.005	1.01(1.00-1.02)	0.18
Vasopressor at ED	2.59(2.32–2.89)	< 0.001	2.50(2.20–2.84)	< 0.001

^(a)^*Abbreviation* IQR, interquartile range; qSOFA, quick Sepsis-related Organ Failure Assessment; ED, emergency department; ICU, intensive care unit

^(b)^ RR, relative risk; CI, confidence interval

Considering the admission to the ICU, association was observed with three points on the severity criteria and higher values of creatinine in the univariate analysis. Scoring any point on qSOFA was considered as an independent factor for ICU admission (qSOFA = 1 point, *p* = 0.02; qSOFA = 2 points, *p* = 0.03, and qSOFA = 3 points, *p* = 0.04) in the multivariate analysis. Positive blood cultures (RR, 1.17; CI 95%, 1.01 to 1.36) and administration of vasopressors in the ED (RR, 2.50; CI 95%, 2.20 to 2.84) were also considered as risk factors when included in the model.

**Fig. 2:**
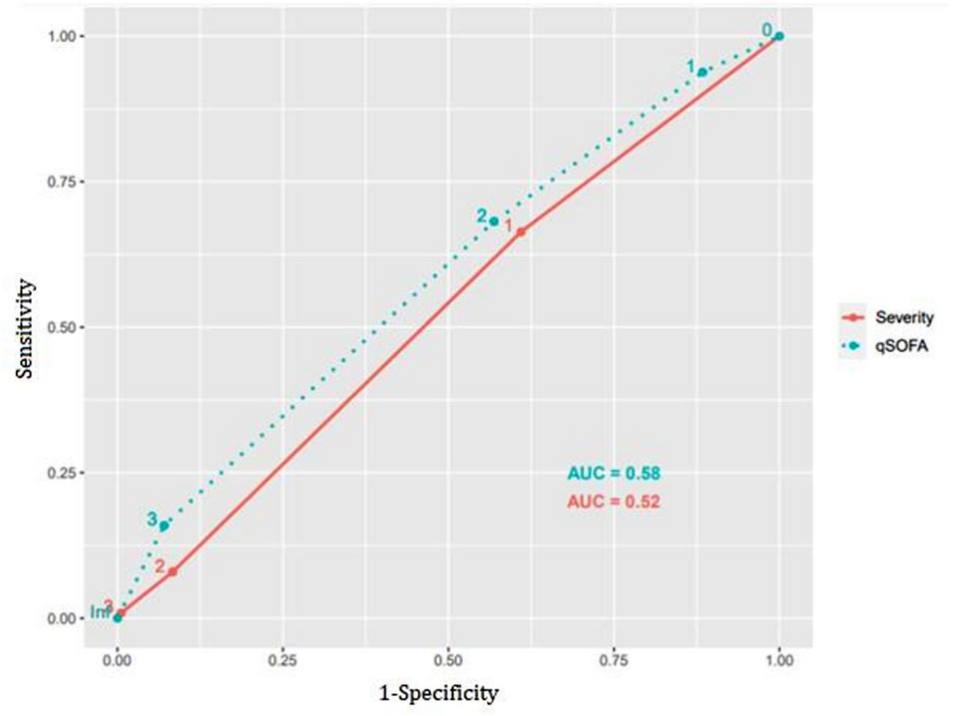
Receiver operating characteristic curves for in-hospital mortality

When evaluating the three different antibiotic administration intervals (less than 30 min, 30–60 min, and more than 60 min) in relation to mortality, no statistical significance was found concerning survival rates (*p* = 0.21). Figure 2 presents two AUROC curves estimating the accuracy of various cutoff points for the main scores analyzed, along with predictions for 30-day mortality. The highest AUROC was found for qSOFA (AUC = 0.58), and the lowest AUROC was found for the severity criteria (AUC = 0.52). A qSOFA score of two or more points was used as the reference baseline, while one or more points were used as the reference baseline for the severity criteria curve. The sensitivity for the qSOFA curve was 20% (95% CI 16%-24%), with a specificity of 87% (95% CI 82%-91%). The sensitivity for the severity criteria curve was 18% (95% CI 15%-22%), with a specificity of 85% (95% CI 80%-89%).

## Discussion

Given that sepsis is a highly heterogeneous clinical syndrome with significant morbidity and mortality, and that early identification directly impacts patient prognosis, it is crucial to develop clinical scores that are easy and quick to use for identifying patients during emergency triage. Currently, we lack an ideal score for this purpose.

The present study has shown that qSOFA and the severity criteria should not be recommended as tools to predict mortality in patients with suspicion of sepsis in the ED triage. However, it can be considered a valuable predictor of mortality for ICU admission. A prospective study has demonstrated that qSOFA failed to be an adequate screening tool for the recognition of sepsis, 30-day mortality, or prolonged ICU stay, although qSOFA was superior compared to systemic inflammatory response syndrome (SIRS) in predicting 30-day mortality [7]. These findings are in line with the results found in our study, although other studies have shown the opposite outcome [17–20]. The present study was carried out in a private hospital in Brazil with a sample of patients that do not represent the general population of the country, since the vast majority of the patients studied represent a middle- and upper-class population. This aspect might explain the divergence of the results of qSOFA with the mortality found in our study in comparison to a similar study performed within a lower income population [19]. These findings suggest that qSOFA may not be an adequate tool to predict mortality in hospitals that care for medium and high-income patients but could be used in institutions with fewer resources. The average age of the patients included in our study was 73 years, which represents an older population with a greater burden of comorbidities and higher rates of the admission in the ICU. Interestingly, this finding was described in a similar study carried out with older patients in a Brazilian private hospital [17].

No association was found between the severity criteria and mortality in this study. Although the severity criteria have not been tested in previous studies, their components are similar to predictors of mortality used in another research. The mottling score used in our study’s severity criteria is comparable to the mottling score that showed an association with 14-day mortality in a previous study [10]. . Shivering, described in the severity criteria and suggesting bacteremia, aligns with findings from another study, which reported higher mortality between 28 and 90 days in patients with positive blood cultures [13]. The cutoff point for tachycardia in the severity criteria (120 bpm) is less sensitive than that used in the SIRS criteria (90 bpm). This difference might influence the sensitivity of SIRS compared to the severity criteria in predicting mortality in patients with suspected sepsis in the ED [21].

In this study, we found a high proportion of gram-negative bacteria among positive blood cultures, with the most common bacteria being *Escherichia coli* (39,8%). Positive blood cultures emerged as a significant risk factor for 30-day mortality (RR, 1.63; CI 95%, 1.10–2.41; *p* = 0.02) and ICU admission (RR, 1.17; 95% CI, 1.01–1.36; *p* = 0,04). In a retrospective cohort study conducted in Beijing Chao-Yang Hospital, *Escherichia coli* was also the most common bacteria detected in blood cultures of 640 patients and was also identified that culture-positive patients were more likely to be admitted to the ICU, to have longer ICU lengths of stay, to present higher clinical severity and higher in-hospital mortality than culture-negative patients [13].

In a meta-analysis published in 2021, whose objective was to review the impact of timelines of ATB administration in the outcome of patients with sepsis and septic shock, two-thirds of studies reported an association between early ATB administration and mortality [22]. The results presented in our study did not show association with any outcomes presented in the aforementioned study. Some of the possible explanations for these results are: (*i*)inadequate reports of the time ATBs were initiated by the staff; (*ii*)not all patients in the course of the hospitalization were considered septic patients, being classified with other alternative diagnoses; (*iii*) the time before ATB administration was different among the several studies considered, contributing to the heterogeneity of results and (iv) some patients admitted with suspected sepsis were already receiving ATBs at home, which could have influenced the evolution of their condition.

This study presents several strengths. First, it evaluates the performance of screening tools (qSOFA and the severity criteria) in the ED, searching for associations among the main adverse outcomes linked to septic patients (30-day mortality, length of stay in hospital, ICU admission, and overall mortality), besides the scores of different age groups. Secondly, the studied sample was relatively large with few missing data. Thirdly, this paper presents an opportunity to test a new tool in the ED to predict adverse outcomes in septic patients at early stages of the condition. Nevertheless, the limitations of the study need equal addressing. First, this is a retrospective study, making it susceptible to several types of bias and unable to guarantee causality among variables. Second, although the study was conducted at a single center, which may limit its generalizability, the center is a tertiary referral hospital in the country. It admits patients with diverse conditions and complexities, making its findings potentially comparable to populations in other countries, such as the United States, and other countries in Europe and Asia. Thirdly, it is possible that patients who registered zero or one point on qSOFA (with no score on the severity criteria at presentation) could have been excluded from the sepsis protocol. Such distortion could have underestimated the real number of patients of interest. Fourthly, there may have been an indication bias related to admission to the ICU, since the hospital has a large number of beds available, and patients with less severity may have been listed without a precise indication of the need for ICU. Finally, alternative diagnoses other than sepsis could have also been addressed at presentation, which provides inputs for future research.

## Conclusions

Although this study did not identify an association between qSOFA and the severity criteria in predicting mortality for patients admitted to the ED, these scores could be useful in resource-limited hospitals. They are simple tools that can ensure greater adherence by the care team and minimize the impact of this pathology in their environment. We found an association between higher qSOFA scores and ICU admission. Therefore, patients with a tendency toward greater severity could be identified and treated more quickly and effectively in the emergency department. This approach could decrease ICU admissions, reducing unnecessary costs and complications linked to the intensive care environment, such as ventilator-associated infections and other related issues. However, prospective studies are necessary to confirm these hypotheses.

## Data Availability

The dataset used and analyzed during the current study is available from the corresponding author on reasonable request.

## References

[1] Fleischmann C, Scherag A, Adhikari NKJ, Hartog CS, Tsaganos T, Schlattmann P (2016). Assessment of Global Incidence and Mortality of Hospital-treated Sepsis. Curr Estimates Limitations.

[2] Reinhart K, Daniels R, Kissoon N, Machado FR, Schachter RD, Finfer S (2017). Recognizing Sepsis as a Global Health Priority — a WHO Resolution. N Engl J Med.

[3] Rudd KE, Johnson SC, Agesa KM, Shackelford KA, Tsoi D, Kievlan DR (2020). Global, regional, and national sepsis incidence and mortality, 1990–2017: analysis for the global burden of Disease Study. Lancet.

[4] Singer M, Deutschman CS, Seymour CW, Shankar-Hari M, Annane D, Bauer M (2016). The Third International Consensus definitions for Sepsis and septic shock (Sepsis-3). JAMA.

[5] Seymour CW, Liu VX, Iwashyna TJ, Brunkhorst FM, Rea TD, Scherag A (2016). Assessment of Clinical Criteria for Sepsis: for the Third International Consensus definitions for Sepsis and septic shock (Sepsis-3). JAMA.

[6] Song JU, Sin CK, Park HK, Shim SR, Lee J (2018). Performance of the quick sequential (sepsis-related) organ failure Assessment score as a prognostic tool in infected patients outside the intensive care unit: a systematic review and meta-analysis. Crit Care.

[7] Loritz M, Busch HJ, Helbing T, Fink K (2020). Prospective evaluation of the quickSOFA score as a screening for sepsis in the emergency department. Intern Emerg Med.

[8] Freund Y, Lemachatti N, Krastinova E, Van Laer M, Claessens YE, Avondo A (2017). Prognostic accuracy of Sepsis-3 criteria for In-Hospital mortality among patients with suspected infection presenting to the Emergency Department. JAMA.

[9] Bone RC, Balk RA, Cerra FB, Dellinger RP, Fein AM, Knaus WA (1992). Definitions for Sepsis and Organ failure and guidelines for the use of innovative therapies in Sepsis. Chest.

[10] Ait-Oufella H, Lemoinne S, Boelle PY, Galbois A, Baudel JL, Lemant J (2011). Mottling score predicts survival in septic shock. Intensive Care Med.

[11] Ait-Oufella H, Joffre J, Boelle PY, Galbois A, Bourcier S, Baudel JL (2012). Knee area tissue oxygen saturation is predictive of 14-day mortality in septic shock. Intensive Care Med.

[12] Phua J, Ngerng W, See K, Tay C, Kiong T, Lim H (2013). Characteristics and outcomes of culture-negative versus culture-positive severe sepsis. Crit Care.

[13] Yang L, Lin Y, Wang J, Song J, Wei B, Zhang X (2021). Comparison of clinical characteristics and outcomes between positive and negative blood culture septic patients: a retrospective cohort study. IDR.

[14] Coudroy R, Jamet A, Frat JP, Veinstein A, Chatellier D, Goudet V (2015). Incidence and impact of skin mottling over the knee and its duration on outcome in critically ill patients. Intensive Care Med.

[15] Dumas G, Lavillegrand JR, Joffre J, Bigé N, de-Moura EB, Baudel JL (2019). Mottling score is a strong predictor of 14-day mortality in septic patients whatever vasopressor doses and other tissue perfusion parameters. Crit Care.

[16] Paquette K, Sweet D, Stenstrom R, Stabler SN, Lawandi A, Akhter M (2021). Neither blood culture positivity nor Time to positivity is Associated with Mortality among patients presenting with severe manifestations of Sepsis: the FABLED cohort study. Open Forum Infect Dis.

[17] Ramos JGR, da Hora Passos R, Teixeira MB, Gobatto ALN, Coutinho RV dos, Caldas S. Prognostic ability of quick-SOFA across different age groups of patients with suspected infection outside the intensive care unit: a cohort study. J Crit Care. 2018;47:178–84. 10.1016/j.jcrc.2018.07.008 30005305

[18] Canet E, Taylor DM, Khor R, Krishnan V, Bellomo R (2018). qSOFA as predictor of mortality and prolonged ICU admission in Emergency Department patients with suspected infection. J Crit Care.

[19] Boillat-Blanco N, Mbarack Z, Samaka J, Mlaganile T, Mamin A, Genton B (2018). Prognostic value of quickSOFA as a predictor of 28-day mortality among febrile adult patients presenting to emergency departments in Dar Es Salaam, Tanzania. Nanayakkara PWB, organizador. PLoS ONE.

[20] Lee J, Song JU (2020). Performance of a quick sofa-65 score as a rapid sepsis screening tool during initial emergency department assessment: a propensity score matching study. J Crit Care.

[21] Fernando SM, Tran A, Taljaard M, Cheng W, Rochwerg B, Seely AJE (2018). Prognostic accuracy of the Quick Sequential Organ failure Assessment for Mortality in patients with suspected infection: a systematic review and Meta-analysis. Ann Intern Med.

[22] Asner SA, Desgranges F, Schrijver IT, Calandra T (2021). Impact of the timeliness of antibiotic therapy on the outcome of patients with sepsis and septic shock. J Infect.

